# Metal Chelates of Petunidin Derivatives Exhibit Enhanced Color and Stability

**DOI:** 10.3390/foods9101426

**Published:** 2020-10-09

**Authors:** Peipei Tang, M. Monica Giusti

**Affiliations:** Department of Food Science and Technology, 2015 Fyffe Ct., The Ohio State University, Columbus, OH 43210, USA; tang.451@buckeyemail.osu.edu

**Keywords:** purple potato (*Solanum tuberosum* L. subsp. *andigenum*), black goji (*Lycium ruthenicum* Murr.), anthocyanin, natural colorant, metal chelation

## Abstract

Anthocyanins with catechol (cyanidin) or pyrogallol (delphinidin) moieties on the B-ring are known to chelate metals, resulting in bluing effects, mainly at pH ≤ 6. Metal interaction with petunidin, an *O*-methylated anthocyanidin, has not been well documented. In this study, we investigated metal chelation of petunidin derivatives in a wide pH range and its effects on color and stability. Purple potato and black goji extracts containing >80% acylated petunidin derivatives (25 μM) were combined with Al^3+^ or Fe^3+^ at 0 μM to 1500 μM in buffers of pH 3–10. Small metal ion concentrations triggered bathochromic shifts (up to ~80nm) at an alkaline pH, resulting in vivid blue hues (h_ab_ 200°–310°). Fe^3+^ caused a larger bathochromic shift than Al^3+^, producing green colors at pH 8-9. Generally, metal ions increased the color stability and half-life of petunidin derivatives in a dose-dependent manner, particularly at pH 8. Petunidin derivative metal chelates produced a wide range of colors with enhanced stability.

## 1. Introduction

Anthocyanins are water-soluble pigments that provide brilliant red, purple and blue colors to vegetables and fruits [1]. They undergo characteristic pH-dependent structural transformations, exerting a diverse color appearance [2]. The consumption of fruits rich in anthocyanins is believed to benefit health and prevent the development of diabetes, cancer, and cardiovascular disease, based on epidemiological studies [3]. Given the fact that they possess potent antioxidant activity and are associated with health-promoting effects, anthocyanins have become an increasingly prevalent alternative to the current widely used synthetic food dyes which are suspected to cause activity problems in children [1,4,5].

**Figure 1:**
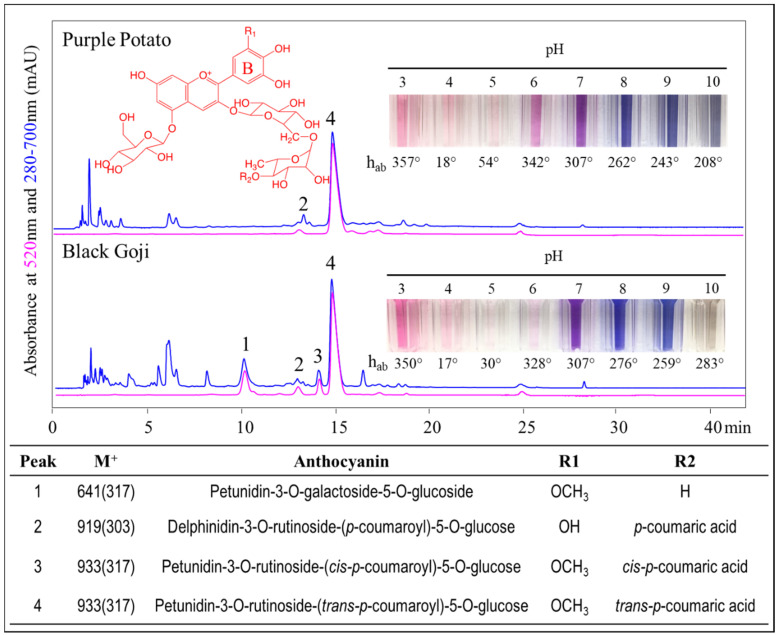
Purple potato and black goji anthocyanin extracts chromatograms at 520 nm and 280–700 nm, and their color expression at different pH.

Anthocyanin color and stability could be influenced by metal complexation [6]. It was reported that anthocyanins with at least two free hydroxyl groups on the B-ring could chelate multivalent metal ions, including, but not limited to, Fe^2+^, Fe^3+^, Ga^3+^, Al^3+^, Mg^2+^, Sn^2+^, Ti^2+^, and Cu^2+^ [7,8,9,10]. The metal ion acts in competition with H^+^ to attach to the catechol or pyrogallol moieties on the B-ring, triggering anthocyanin transformation from a red flavylium cation form to a purple-blue quinoidal base anion form. This transformed molecule could then stack with other flavylium cation molecules to form a stable complex, leading to a bathochromic shift in the spectrum and a bluing effect [11]. Although various types of anthocyanin–M^n+^ complexes exhibiting blue colors have been reported, most of these studies mainly focused on cyanidin (two hydroxyl groups on the B-ring) and delphinidin derivatives (three hydroxyl groups on the B-ring) in acidic or mildly acidic conditions (pH ≤ 6) [8,11,12,13,14,15]. A recent study demonstrated that peonidin and cyanidin derivatives from purple sweet potato could bind Fe^2+^ and Al^3+^ via the anthocyanin chromophore and caffeoyl residues at a neutral pH [16]. Besides, ion traces were found to initiate caffeoyl residue autoxidation, leading to color loss, while anthocyanins carrying two caffeoyl residues were relatively invulnerable to the Fe^2+^ pro-oxidation effect due to a rigid iron chelation [16]. Anthocyanin–M^n+^ chelation has been studied in foods such as strawberry puree [17], cranberry juice cocktail [6], crowberry juice [6], and in food model systems with sugar beet pectin [18], isolated pectin fractions [18], and polysaccharides [12] as a practical method to stabilize anthocyanin color. Metal chelation of anthocyanins has been reported to cause bathochromic shifts and favor anthocyanin expression of blue colors in cyanidin-, delphinidin-, and peonidin-rich materials [15,19]. To our knowledge, metal chelation with petunidin derivatives has not been previously reported. It remains to be explored if the metal chelation and the bluing effect could be observed in petunidin derivatives which possess two hydroxyl groups and one methoxy group on the B-ring (Figure 1). It is important to the food industry, as the elucidation of the anthocyanin–metal interaction would bring innovative technologies to obtain a wide range of vivid colors from natural sources, fulfilling the current clean label trend.

Purple potato (*Solanum tuberosum* L. subsp. *andigenum*) and black goji (*Lycium ruthenicum* Murr.) are two sources rich in petunidin derivatives. Purple potato has been increasingly popular among consumers during the past few years because of its potential health benefits [20]. Compared to white and yellow flesh potatoes, purple potato contains plentiful polyphenols, anthocyanins, and phenolic acids [21]. There are up to 1.5 g anthocyanins/kg DW in purple potato, and petunidin derivative was the predominant (~63%) pigment [20]. An average of 4.68 g of gallic acid (GA) equivalent polyphenols per kg DW were found, 3–4-folds of that in white flesh cultivars [20,22]. The Chinese herb black goji commonly grows on the Qinghai–Tibet Plateau. Its fruit, black goji berry, contains abundant anthocyanins (up to 5.5 g/kg FW) [3,23,24]. There are four major anthocyanins reported in the literature, and petunidin derivatives were the predominant (95%) pigments [3,23,24]. Black goji berry is rich in polyphenols (~13.1 g GA equivalents/kg FW) and possesses strong antioxidant activity (~10.60 g GA equivalents/kg FW) [23]. Our previous study showed that the petunidin-3-*trans*-*p*-coumaroyl-rutinoside-5-glucoside isolate contributed most of the color expression of the black goji extract. It also showed superior stability compared to crude extracts over time [24,25].

This study aimed to investigate the colorimetric and spectrophotometric properties of petunidin derivatives from purple potato and black goji, and to examine the impact of their metal chelation on the color expression and stability. We hypothesize that the metal chelation and the bluing effect can be observed in petunidin derivatives. This study adds to our knowledge of anthocyanin–metal chelation and provides the food industry with additional alternatives for producing a wider variety of colors derived from nature.

## 2. Materials and Methods

### 2.1. Materials & Reagents

Pigments were extracted from purple potatoes (cultivar Purple Majesty) purchased from a local grocery store (Whole Foods Market) in Columbus, Ohio, and black goji berries were obtained from LianHua Supermarket in Shanghai, China.

The chemicals and reagents used were obtained from Fisher Scientific (Fair Lawn, NJ, USA) as either ACS or HPLC grade: acetone, chloroform, methanol, citric acid, Na_2_HPO_4_, NaH_2_PO_4_, Na_2_CO_3_, NaHCO_3_, KCl, CH_3_COONa, lab-grade aluminum sulfate hydrate, reagent-grade ferric chloride hexahydrate, and LC/MS-grade acetonitrile. In addition, ACS grade ethyl acetate (C_4_H_8_O_2_) and formic acid (HCO_2_H) were acquired from Mallinckrodt Chemicals (Bedminster Township, NJ, USA) and Honeywell (Morris Plains, NJ, USA), respectively.

### 2.2. Pigment Extraction

Purple potato and black goji pigments were extracted by using acetone, and partitioned with chloroform. Approximately 100 g purple potato or black goji berry were sliced and mingled with liquid nitrogen before being powdered in a food blender. Acidified acetone (0.01% HCl) was blended with the powders, and then the pigment slurry was filtered through Whatman No. 4 filter paper (Whatman Incorporation, Florham Park, NJ, USA). Re-extraction was conducted using 70% (*v*/*v*) acidified (1% HCl) aqueous acetone that was used to re-wash the slurry until the filtrate was clear. The filtrate was then transferred to a separatory funnel, and gently mixed with chloroform. After that, the separatory funnel was stored in refrigerated dark conditions overnight, so that the pigments were concentrated in the upper aqueous phase. It was collected the next day in a round flask to remove the acetone and chloroform by a rotary evaporator.

### 2.3. Pigment Purification

The purification of pigment crude extracts was performed based on the method proposed by [26]. The aqueous anthocyanin extracts were forced to pass through a Sep-Pak^®^ C18 cartridge (Waters Corporation, Milford, MA, USA) which had been activated with methanol. Two volumes of acidified water (0.01% *v*/*v* HCl) were then pumped to wash the column, followed by one volume of ethyl acetate. Next, the purple potato or black goji anthocyanins were recovered by flushing the column with acidified methanol (0.01% *v*/*v* HCl) and collected in a round flask. After the removal of methanol in a rotary evaporator at 40 °C, the purified anthocyanin was dissolved in acidified distilled water (0.01% *v*/*v* HCl).

### 2.4. Pigment Identification

Anthocyanins from purple potato and black goji extracts were identified by high-performance liquid chromatography (HPLC) (Shimadzu, Columbia, MD) using an SP-M20A photodiode array detector and an LCMS-2010EV liquid chromatograph mass spectrometer. A reverse phase Symmetry C-18 column with 5μm particle size and 4.6 × 150 mm column size (Phenomenex, Torrance, CA, USA) was used to separate pigments. Extracts were forced through a 0.22 um syringe filter (Phenomenex, Torrance, CA, USA) before HPLC injection. The mobile phase was solvent A (4.5% (*v*/*v*) formic acid) and solvent B (100% acetonitrile). The flow rate was set to be 0.8 ml/min. The linear gradient in this study was programmed as follows: 7% B 0–1 min, 7–20% B 1–30 min, 20–40% B 30–36 min, 40% B 36–40 min, 40–7% B 40–42 min, 7% B 42–52 min. Anthocyanin and phenolic compound elutions were monitored at 500–530 nm and 280–700 nm, respectively. Total ion scan and selected ion monitoring (positive mode with mass/charge ratio of 271, 287, 303, 301, 317, and 331, corresponding to the six most common anthocyanin aglycones) were used to analyze the pigment composition in samples.

### 2.5. Pigment Quantification

The monomeric anthocyanin content was quantified by the pH differential method adopted from [27]. Purified anthocyanin samples were dissolved in 0.025 M potassium chloride buffer (pH 1) and 0.4 M sodium acetate buffer (pH 4.5). After 15 min equilibrium, the absorbances at λ_vis-max_ (524 nm) and at 700 nm were measured by a UV–Vis spectrophotometer (Shimadzu Corporation, Tokyo, Japan). The quantification was accomplished in triplicate, and the monomeric anthocyanin contents of the extracts were expressed as cyanindin-3-glucoside equivalents (MW = 449.2, molar absorptivity = 26,900). Anthocyanin purity was defined as: the area under the curve (AUC) at 520 nm / AUC at max absorbance at 280–700 nm in the HPLC chromatograph.

### 2.6. Buffer System and Sample Preparation

A series of buffer solutions ranging from pH 3 to 10 were prepared (citric acid-Na_2_HPO_4_ buffer solutions for pH 3–7; Na_2_HPO_4_-NaH_2_PO_4_ buffer solution for pH 8; Na_2_CO_3_-NaHCO_3_ buffer solutions for pH 9–10) [28]. Purple potato and black goji anthocyanin extracts (25 μM) were diluted in the above buffers and the final mixtures’ pH environment was confirmed by a pH meter (Mettler Toledo Inc., Columbus, OH, USA). For the metal chelation study, metal ion solutions (Al^3+^ or Fe^3+^) were prepared at a concentration of 0.06 M before mixing with 25 μM purple potato or black goji anthocyanin extracts based on specific target anthocyanin to metal ion molar ratios (1:0.1, 1:0.5, 1:1, 1:2, 1:5, 1:10, 1:30, 1:60) in buffers of pH 3–9 (namely, the metal ion concentrations were 2.5, 12.5, 25, 50, 125, 250, 750, and 1500 μM). The final pH condition in each combination was also confirmed by a pH meter. Although the pH differential method is a widely used method for quantifying anthocyanins, the absorbance maximum and molar extinction coefficient for cyanidin 3-O-glucoside are much different than those of the petunidin anthocyanins presented here. Therefore, the “molar ratios” reflect the relative amount of anthocyanins (in cyanindin-3-glucoside equivalents) and metal ions.

The initial spectrophotometric and colorimetric measurements were collected after 60 min of equilibration. Anthocyanin samples chelated with metal ions were stored in refrigerated (4 °C) conditions in the dark for 4 weeks for the stability test. Experiments were done in triplicate.

### 2.7. Spectrophotometric Analysis

Anthocyanin samples with or without metal ions were mixed with different buffer systems before being transferred (250 μL) to poly-D-lysine-coated polystyrene 96-well plates. The spectra of these samples from 380 to 700 nm (1nm interval) were collected by using a SpectraMax 190 Microplate Reader (Molecular Devices, Sunnyvale, CA, USA). Samples were sealed in a well plate with parafilm to prevent evaporation, and were stored refrigerated (4 °C) in the dark.

### 2.8. Half-Life Calculation

The absorbance at the *λ*_max_ from *t*_0_ (60 min) of the sealed samples (described in Section 2.7. Spectrophotometric Analysis) were measured at 60 min and at 1, 2, and 3 days. Linear regressions (*R*^2^ > 0.8) were modeled from the natural logarithm of the absorbances at each time point based on the formula ln(At) = −kt + ln(A_60min_). The half-lives were calculated as t_1/2_ = ln2/k.

### 2.9. Colorimetric Analysis

The color characteristics of each mixture were measured by using a Hunter ColorQuest XE spectrophotometer (HunterLab, Reston, VA, USA) using total transmission mode; the data were presented in a CIELab system (L*, C*_ab_, h_ab_, a*, and b*) using a 10° observer angle and D65 illuminant spectral distribution. Color was monitored over 4 weeks under refrigerated storage in the dark (60 min after mixing, and on day 1, 2, 3, 4, 5, 7, 14, 21, and 28). The color changes were calculated as ΔE*_ab_ using the 60 min colorimetric data as a control (formula is listed below). All the samples were sealed in cell culture flasks throughout the study.

(1) $${\Delta E}_{\mathrm{ab}}^{*}=\sqrt{{\left(L_{t}^{*}-L_{60\min}^{*}\right)}^{2}+{\left(a_{t}^{*}-a_{60\min}^{*}\right)}^{2}+{\left(b_{t}^{*}-b_{60\min}^{*}\right)}^{2}}$$

### 2.10. Statistical Analysis

Statistical analysis was conducted by Prism software (GraphPad, La Jolla, CA, USA). One-way ANOVA (two-tailed, α = 0.05) was conducted to compare the differences among each treatment group. In case of significance, a post hoc Tukey’s test with family-wise α = 0.05 was performed.

## 3. Results and Discussion

### 3.1. Anthocyanin Profiles in Purple Potato and Black Goji

Anthocyanins from purple potatoes (cultivar Purple Majesty) and black goji were separated as shown in Figure 1, with 82% and 70% purity, respectively. In purple potato, one anthocyanin accounted for 91% of the total peak area at 520nm and was identified as petunidin-3-rutinoside-(*p*-coumaroyl)-5-glucose (peak 4, Figure 1). A second minor peak (peak 2) representing ~4% of the total peak area was delphinidin-3-rutinoside-(*p*-coumaroyl)-5-glucoside. The same pigments were identified in purple potato varieties by previous researchers [20,29,30] although their proportions varied widely.

Three major black goji anthocyanins accounted for 96% of the total peak area at 520nm (Figure 1). The peak areas of these pigments (peaks 1, 3, and 4) were approximately 18%, 7%, and 71%, respectively. According to the MS data and acid hydrolysis, all major anthocyanins in black goji were petunidin derivatives and ~80% of the total pigments were acylated. The minor peak (peak 2) was identified as a delphinidin derivative and represented less than 5% of the anthocyanins. The pigments were petunidin-3-galactoside-5-glucoside (peak 1), delphinidin-3-*trans*-*p*-coumaroyl-rutinoside-5-glucoside (peak 2), petunidin-3-*cis*-*p*-coumaroyl-rutinoside-5-glucoside (peak 3), and petunidin-3-*trans*-*p*-coumaroyl-rutinoside-5-glucoside (peak 4), agreeing with a previous study [23]. Interestingly, two black goji anthocyanins (peaks 2 and 4) matched two of the pigments found in purple potato.

The extracts were purified by a C18 cartridge with a wash of ethyl acetate, which removed the less polar phenolic compounds [26]. By using the max absorbance at 280–700 nm and mass spectrometry data and comparing to previous references [20,31,32], we have identified other phenolics, such as 5-*O*-caffeoylquinic acid (355 *m*/*z*), 4-*O*-caffeoylquinic acid (355 *m*/*z*), and caffeic acid (181 *m*/*z*) in purple potato, and dihydrocaffeoyl-caffeoyl spermidine derivatives in black goji. Our previous study showed no significant differences in the colorimetric and spectrophotometric properties between the black goji crude extracts and C-18 cartridge-purified extracts [24]. Therefore, the anthocyanin purity in that study did not affect the color considerably.

### 3.2. Spectrophotometric and Colorimetric Properties of Purple Potato and Black Goji Extracts

Generally, both extracts displayed vivid red, purple, and violet-blue hues in acidic, neutral, and alkaline pH, respectively (Figure 1). Anthocyanins undergo pH-dependent structure transformation [2,33], and under acidic pH, the flavylium cation predominates. Purple potato and black goji extracts exhibited hue angles (h) around 355° in CIE L* C*_ab_ h_ab_ color space at pH 3, corresponding to red-purple hues (Table 1). When the pH increases to a mildly acidic environment, hydration of the flavylium cation is expected with the formation of the colorless carbinol pseudobase form. As a result, at pH 4 and 5, the chroma (C*_ab_) decreased and the color of the extracts became pale and dull (Table 1 and Figure 1). At neutral pH, the quinoidal base is formed, and it starts to carry negative charges as the pH reaches more alkaline conditions, leading to blue colors. The hue angles of the purple potato extracts turned from 341.6° at pH 6 to 207.8° at pH 10. Similarly, black goji extracts experienced the same changes in hue angles, but to a lesser degree (327.7° at pH 6 to 282.6° at pH 10). Remarkably, petunidin derivatives displayed various vivid blue hues in neutral and alkaline conditions, with corresponding hue angles ranging from 242.6° and 306.6° (Table 1). The chroma of both extracts also was enlarged in these neutral–alkaline pHs.

**Figure 2:**
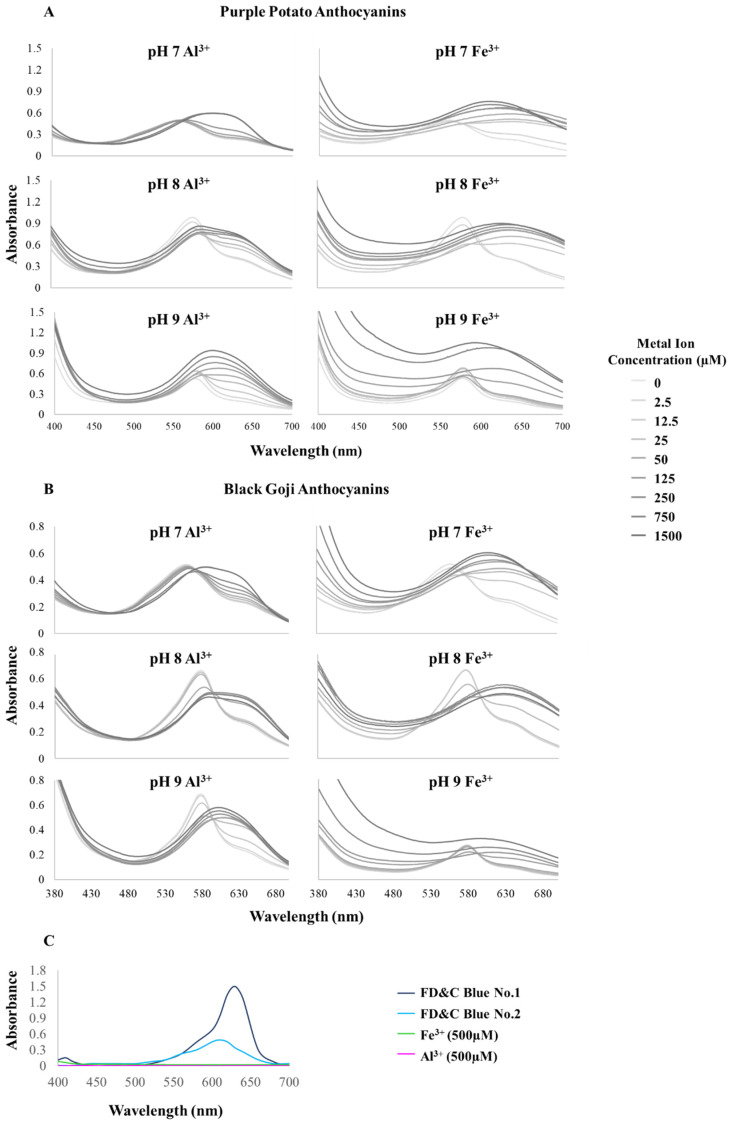
Spectral characteristics of purple potato (**A**) and black goji (**B**) anthocyanin metal chelates at neutral and alkaline pH. The control groups including FD&C Blue colorants and metal ions (500 μM) were shown in (**C**). Data were collected 1 hr after the anthocyanins were mixed with metal ions and pH adjustment with the buffers. The spectra at acidic pHs are not presented due to no impact of metal addition.

The increase in pH and the resulting color changes toward the blue region were a result of the bathochromic changes in the visible spectra of the pigments (Table 1). The λ_max_ of both extracts at acidic pH was ~524 nm and it was boosted to 578 nm at pH 8 and 9. The visible spectra of purple potato and black goji extracts at alkaline pH presented a distinctive peak shoulder (~630 nm) next to the λ_max_ (Figure 2).

### 3.3. Colorimetric Properties of Petunidin-Rich Extracts after Metal Addition

**Figure 3:**
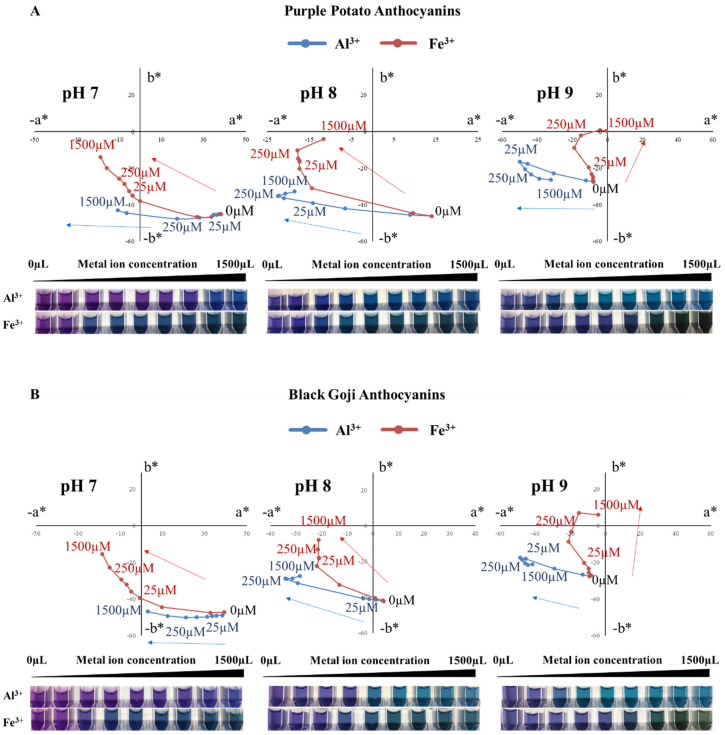
Colorimetric changes of the purple potato (**A**) and black goji (**B**) anthocyanin extracts chelated with metals. Data were collected 1 hr after the anthocyanins were mixed with metal ions and pH adjustment with the buffers.

Bluing responses with vivid colors were found in the petunidin–M^3+^ chelates (abbreviated as Pt-M^3+^) (Figure 3). The color hues of Pt-M^3+^ were dependent upon the metal ion source, pH, and Pt-M^3+^ ratio.

With the increase in [Al^3+^], purple potato extracts developed a variety of blue colors. At pH 7, brilliant blue with a hue angle of ~261° appeared, starting from [Al^3+^] of 750 μM. Darker blue (~240°) and greenish blue (~200°) were obtained when [Al^3+^] was 50 μM at pH 8 and pH 9, respectively. Fe^3+^ also triggered bluing shifts, as the increasing [Fe^3+^] led to the transition of Pt-Fe^3+^ from quadrant IV (purple to blue region) to quadrant III (green to blue region) in the CIELAB color space (Figure 3). Larger concentrations of Fe^3+^ resulted in the expression of a yellow hue, likely due to oxidation. These yellow tones mixed with the blue colors, producing green colorations when [Fe^3+^] exceeded 750 μM, with hue angles of ~216°, 210°, and 167° at pH 7, 8, and 9, respectively (Figure 3). Similar results were found with black goji Pt-M^3+^ chelates, where various blue and greenish blue colors were obtained in neutral and alkaline pH (Figure 3).

The ratio between petunidin derivatives and M^3+^ needed for blue color formation was closely related to the pH of the buffer and M^3+^ source. Less [Al^3+^] was required to induce blue hue formation when the pH increased from 7 to 9, supporting the theory of the competition between metal ions and hydrogen ions for attachment to B-ring catechol moiety [34]. However, increasing [Fe^3+^] was essential for the blue-green hue development regardless of pH. This could be due to the aggregation and precipitation of anthocyanin pigment by the excess addition of Fe^3+^, as well as the poor solubility of Fe^3+^ in alkaline pH [8,16,18,35].

In order to measure the minimum [M^3+^] necessary to trigger a color change in purple potato and black goji extracts, ΔE*_ab_ was calculated. Typically, ΔE*_ab_ = 5 is believed to be noticeable by untrained observers. Both metal ions tested at all pH values were able to cause color changes in Pt-colored solutions even when [M^3+^] was less than or equal to 25 μM. As the pH increased from 7 to 9, Pt-M^3+^ required less [Al^3+^] to achieve a noticeable color change (ΔE*_ab_ ≥ 5), while more [Fe^3+^] was needed. In purple potato extracts, Al^3+^ in small concentrations ([Al^3+^] = 12.5 μM) could produce blue colors at pH 8 and 9, and it was the same for Fe^3+^ at pH 7 and 8 (Figure 3 and Table 2). These low-threshold properties are desirable since there are some concerns regarding the neurotoxicity associated with metals [36]. However, both aluminum sulfate (21 CFR section 182.1125) and ferric chloride (21 CFR section 184.1297) are considered by the FDA as Generally Regarded as Safe (GRAS), with no limits to usage. In addition, a metallic salt is an important ingredient in FD&C lake pigments, e.g., Red 40 Aluminum Lake and Blue #1 and #2 Aluminum Lake. The metal ion concentrations used in this study were lower than those in FD&C lake pigments. Therefore, the Pt-M^3+^ could be a potential method to decrease metal usage in food pigments in neutral and alkaline pH.

At pH 3–6, however, Pt-Al^3+^ and Pt-Fe^3+^ ([M^3+^] was up to 1500 μM) seemed to have limited impact on Pt colorimetric properties, as the ΔE*_ab_ were all smaller than 5 (data not shown). In addition, the color of petunidin derivatives faded quickly at pH 10. Therefore, our following analysis focused on the Pt-M^3+^ at pH 7–9.

### 3.4. Spectrophotometric Properties of Petunidin Derivatives Chelated with Metals

**Figure 4:**
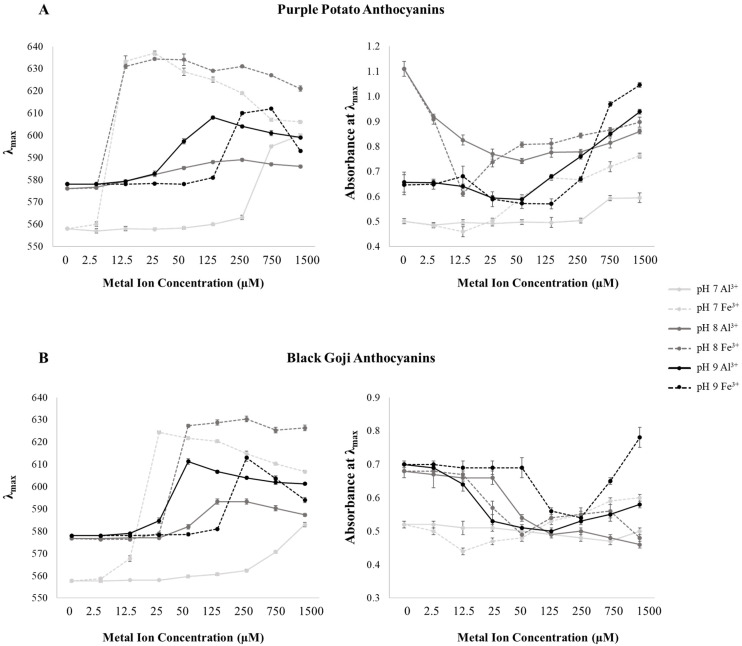
Quantification of spectrophotometric changes in purple potato (**A**) and black goji (**B**) anthocyanin metal chelates. Data were collected 1 hr after the anthocyanins were mixed with metal ions and pH adjustment with the buffers.

The visible absorbance spectra, λ_max_, and absorbance at the λ_max_ of Pt-M^3+^, are shown in Figure 2 and Figure 4. Generally, bathochromic and hyperchromic shifts were perceived for Pt-M^3+^ at pH 7–9, similar to the findings in previous studies on cyanidin or delphinidin–M^3+^ chelates at acidic pHs [8,15,17,35]. Pt-M^3+^ chelation was greatly influenced by pH and metal ion source.

The addition of Al^3+^ to purple potato petunidin derivatives introduced the most pronounced bathochromic shift (Δλ_max_ = 42 nm) at pH 7 and the maximum hyperchromic response (ΔA = 0.282) at pH 9, while Fe^3+^ chelates experienced their maximum shifts in larger magnitudes (Δλ_max_ = 79 nm and ΔA = 0.399, respectively) at the same pH as that of Al^3+^ (Figure 4). Black goji petunidin derivatives exerted the maximum bathochromic shifts with Al^3+^ (Δλ_max_ = 33 nm) and Fe^3+^ (Δλ_max_ = 66 nm) at pH 9 and 7, respectively (Figure 4). Additionally, in purple potato extracts, the largest λ_max_ and absorbance achieved by Pt-Al^3+^ were 609 nm and 0.939 at pH 9, respectively; and Pt-Fe^3+^ reached its maximum λ_max_ (637 nm) and absorbance (1.046) at pH 7 and pH 9, respectively. In black goji extracts, the maximum λ_max_ of Pt-M^3+^ was 611 nm (Pt-Al^3+^) and 630 nm (Pt-Fe^3+^). Overall, Pt-Fe^3+^ showed higher bathochromic and hyperchromic responses than Pt-Al^3+^, similar to previous reported results for cyanidin-M^3+^ [8]. It was proposed by Sigurdson et al. (2016) that the outer electrons of Fe^3+^ appeared to be in a high spin configuration (with one electron in each 5d orbital), resulting in an enhanced overlapping with anthocyanin and a stronger association. This unique electron arrangement was expected to be responsible for the pronounced shifts in visible spectra. Thus, Fe^3+^ in a lower concentration was able to produce a large bathochromic response.

The [M^3+^] that resulted in maximum λ_max_ and absorbance at each pH level was closely related to the M^3+^ source. In purple potato extracts, the largest λ_max_ of Pt-Al^3+^ was accomplished when [Al^3+^] equaled 1500 μM, 250 μM, and 125 μM at pH 7, 8, and 9, respectively; while it was 25 μM, 50 μM, and 750 μM in Pt-Fe^3+^ at these same pH levels (Figure 4). Similar trends could be found in black goji extracts (Figure 4). Actually, the [Al^3+^] that produced the maximum λ_max_ dropped as pH increased, aligning with the idea that M^3+^ acts in competition with H^+^ for B-ring attachment in anthocyanins [34]. However, the level of [Fe^3+^] that produced the largest λ_max_ increased when pH increased, which might be explained by the aggregation and precipitation of anthocyanin pigment triggered by the addition of Fe^3+^, as well as the poor stability of Fe^3+^ at an alkaline pH [8,16,18,35].

Another interesting phenomenon was that when [M^3+^] increased, we observed a hypsochromic effect after the bathochromic effect, indicating an apparent saturation at the turning point (Figure 4). However, this apparent saturation was not found in Pt-Al^3+^ at pH 7, which could suggest that the concentration of [Al^3+^] tested in this study was not high enough to reach saturation. Similarly, the hypochromic response appeared before the hyperchromic effect and the turning point in λ_max_ corresponded to that in absorbance (Figure 4).

Fe^3+^ in high concentrations expressed yellow colors in aqueous systems, documented by the higher absorbance in the spectral region of 380-450 nm. Similarly, Pt-Fe^3+^ gradually developed higher absorbance in a [Fe^3+^] dose-dependent manner in this region at pH 7–9 (Figure 2 and Figure 4). A combination of the blue color from petunidin derivatives and the bluing effect introduced by metal chelation with the yellow hues of Fe^3+^ resulted in a greenish blue color (Figure 3) despite the high absorbance around 610 nm (more typical of blue hues).

### 3.5. Impact of Metal Chelation on the Stability of Petunidin Derivatives

**Figure 5:**
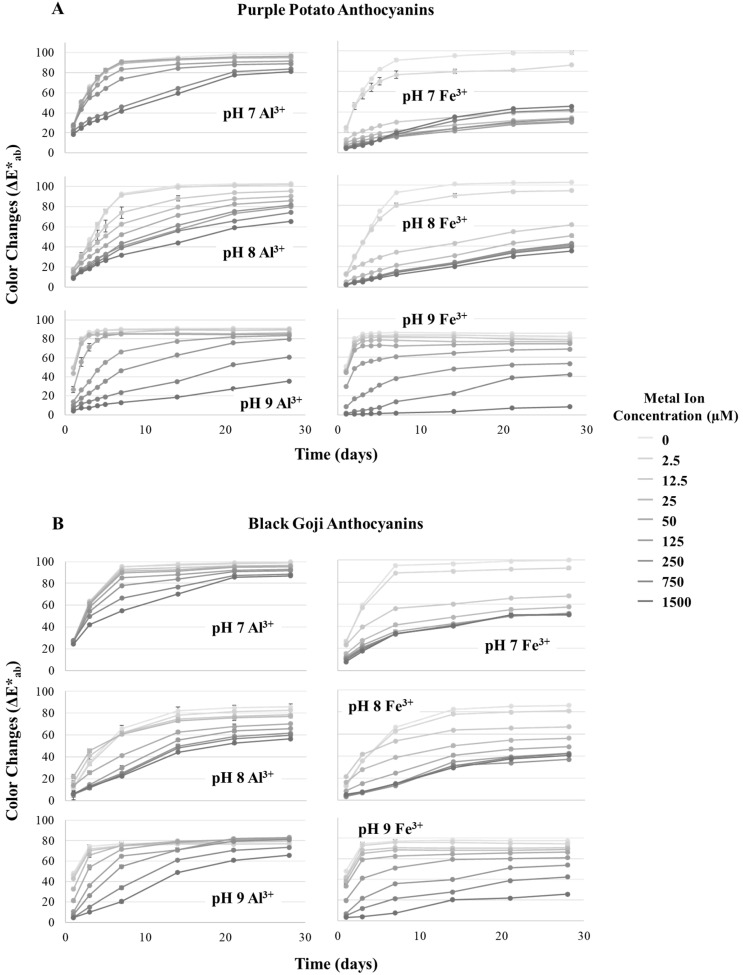
Color changes (ΔE*_ab_) of purple potato (**A**) and black goji (**B**) anthocyanin metal chelates at pH 7–9 over 28 days. Samples were stored under refrigerated conditions in the dark.

**Figure 6:**
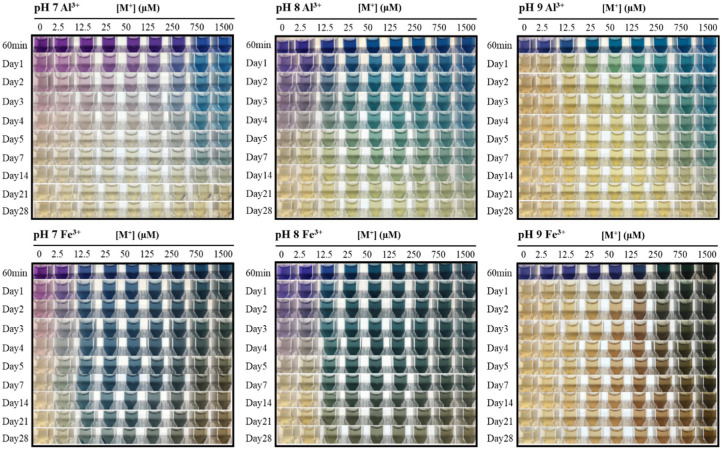
Color stability of purple potato anthocyanin metal chelates at pH 7–9 over 28 days. Samples were stored under refrigerated conditions in the dark.

Pt-M^3+^ was stored in a dark refrigerated condition for 28 days. The color changes expressed as total color change (ΔE*_ab_) are shown in Figure 5 and Figure 6. Pt-M^3+^ generally exhibited enhanced color stability in a [M^3+^] dose-dependent manner. Higher [Al^3+^] produced bluer hues that were significantly better retained over time for ~7 days at all pH values tested (pH 7–9), while petunidin derivatives without metal chelation started losing color rapidly, with the blue color only formed at pH 9 and gone in less than 1 day. Interestingly, brown hues steadily formed in higher [Fe^3+^] chelates after mixing with petunidin derivatives and were more severe at pH 9. Fe^3+^ is known for its reductive–oxidative capabilities, and has been reported to catalyze the oxidative degradation of flavonoids [37]. Fenger et al. reported that anthocyanin caffeoyl residue autoxidation is initiated by Fe^2+^, forming electrophilic/oxidizing *o*-quinones at a neutral pH [16]. They also showed that free caffeoylquinic acids could facilitate the anthocyanin degradation. Although there were no caffeoyl residues in the petunidin derivatives in this study, the surrounding free caffeoylquinic acids in the extracts were expected to promote the autoxidation. Therefore, the brown color could have developed due to the degradation of anthocyanin, especially in the alkaline pH range. In addition, Fe^3+^ in large concentrations expressed yellow-brown colors, which could mask the blue color produced by purple potato anthocyanins, exhibiting green hues. However, when the blue color vanished, the yellow hues would dominate the mixture, which would also partially explain the browning phenomenon.

The half-lives (h) of the pigments and their metal chelates at pH 7–9 were calculated and are presented in Table S1. Similar to ΔE*_ab_, metal ions increased the pigment half-life in a dose-dependent manner over time, particularly at pH 8 (144 h in [Al^3+^] = 1500 μM and 98.5 h in [Fe^3+^] = 50 μM). Because of its reductive–oxidative capabilities, excessive Fe^3+^ started to reduce the half-life of Pt-Fe^3+^ chelates at pH 7–9.

Among the pH values (pH 7–9) in the stability study, petunidin derivatives and their metal chelates displayed the highest color stability at pH 8. At this pH, a lower concentration of M^3+^ still showed a promising stabilization capability. At neutral and alkaline pH, anthocyanin is supposed to exist predominantly in quinoidal base forms, and would take on increasingly negative charges with further increases in pH [38]. This process could be determined by pK_a2_ and pK_a3_, the disassociation constant for the transition from the quinoidal base configuration to that with one negative charge, and the transformation from one negative charge to two negative charges. A previous study reported that the pK_a2_ and pK_a3_ of petunidin aglycone were 6.99 and 8.27, respectively [39]. Although the true pK_a_ for petunidin-3-rutinoside-(*p*-coumaroyl)-5-glucose would be different, it is reasonable to postulate that the purple potato anthocyanin carries negative charges at pH 8 and thus is more stable, compared to other close neutral and alkaline pHs.

Overall, metal ions helped to stabilize petunidin derivatives in a dose-dependent manner, and significantly slowed down the escalation of ΔE*_ab_ and increased the pigments half-life, though this stabilization effect is dependent on the metal source and pH. Fe^3+^ in high concentrations, because of its oxidative capability, accelerated the color degradation and promoted the browning effect.

## 4. Conclusions

Purple potato and black goji extracts contained abundant petunidin derivatives, with petunidin-3-(*trans*-*p*-coumaroyl)-rutinoside-5-glucoside as the predominant pigment. The extracts displayed vivid red, purple, violet-blue colors from acidic, neutral, to alkaline pH, respectively. In the presence of metal ions, Al^3+^ and Fe^3+^, the petunidin derivatives experienced bathochromic (up to 79 nm) and hyperchromic shifts (increased up to 0.399 in absorbance at λ_max_), resulting in various blue to green hues and intensified colors in neutral and alkaline conditions. The magnitudes of the metal chelation effect were dependent on metal ions, pH, and M^3+^ concentration. Fe^3+^, with a higher spin configuration in the outer electrons, triggered further bathochromic shifts than Al^3+^, and its unique yellow color in the aqueous system led to green hues when combined with petunidin derivatives’ blue colors. The oxidative capability of Fe^3+^ towards flavonoids limited its application in larger concentrations (>125 μM). Al^3+^, in low concentrations (~12.5 μM), was able to chelate Pt and produce blue colors at pH 8 and 9. The concentration of Al^3+^ needed for blue color production decreased upon the increase in pH. The chelation of metal ions significantly enhanced the color stability of petunidin derivatives in neutral and alkaline conditions.

This study demonstrated that petunidin derivatives could chelate with metal ions, Al^3+^ and Fe^3+^, in neutral and alkaline pH, resulting in enhanced color and stabilities. Purple potato and black goji, containing abundant petunidin derivatives, are promising sources for natural colorants over a wide range of pH. Chelated with metals, these pigments could provide the food industry with various vivid violet, blue, and green colors with enhanced stability. They could be potentially applied in food products with neutral or slightly higher pH, such as milk, milkshakes, ice cream, and creamers. This is important to the food industry, as the elucidation of the anthocyanin–metal interaction can bring innovative technologies to obtain a wide range of vivid colors from natural sources, fulfilling the current clean label trend.

## Figures and Tables

**Table 1:** Colorimetric (CIE-L*, C*_ab_, h_ab_) and spectrophotometric (λ_vis-max_) data of purple potato and black goji anthocyanin extracts over pH 3–10. Data are presented as means (*n* = 3) followed by standard deviations (in parentheses). NM: not measurable due to color loss.

	pH 3	pH 4	pH 5	pH 6	pH 7	pH 8	pH 9	pH 10
			Purple Potato					
L*(lightness)	91.5 (0.6)	93.0 (0.9)	94.6 (0.3)	90.4 (0.4)	80.2 (1.6)	75.7 (0.5)	74.4 (0.9)	80.8 (2.2)
C*_ab_(chroma)	8.6 (1.2)	5.0 (1.5)	2.6 (0.1)	6.9 (0.8)	17.7 (2.1)	18.8 (0.7)	18.6 (1.2)	7.8 (1.7)
**h_ab_(hue)**	357.3 (3.0)	17.8 (1.2)	54.3 (1.7)	341.6 (4.3)	306.5 (0.4)	262.2 (1.9)	242.6 (3.4)	207.8 (5.6)
**λ_vis-max_**	524 (0.0)	524 (0.0)	529 (0.6)	539 (0.0)	558 (0.0)	577 (0.0)	578 (0.0)	578 (0.0)
			**Black Goji**					
L*(lightness)	90.4 (3.1)	93.0 (1.1)	93.6 (1.1)	81.9 (1.1)	70.2 (0.1)	68.1 (1.2)	69.7 (1.4)	77.1 (1.1)
C*_ab_(chroma)	11.4 (0.9)	5.0 (0.5)	3.7 (1.1)	39.7 (1.7)	40.7 (1.4)	36.4 (1.1)	24.8 (1.9)	27.7 (1.1)
**h_ab_(hue)**	350.2 (2.4)	16.5 (1.8)	30.1 (2.4)	327.7 (1.7)	306.6 (1.7)	275.6 (1.4)	259.3 (1.8)	282.6 (1.4)
**λ_vis-max_**	525 (0.7)	527 (0)	536 (0.7)	539 (1.4)	558 (0.7)	578 (0)	578 (0)	NM

**Table 2:** Color changes (ΔE*_ab_) of the petunidin derivatives with increased metal ion concentration in alkaline conditions (pH 7–9) after 60 minutes of equilibration. Data are presented as the mean and standard deviations (in parentheses).

				Purple Potato Extract					
				[M^3+^]					
		2.5 μM	12.5 μM	25 μM	50 μM	125 μM	250 μM	750 μM	1500 μM
	pH 7	1.4 (0.2)	2.3 (0.3)	3.6 (0.2)	4.8 (0.5)	10.4 (0.2)	20.9 (0.5)	44.8 (1.5)	49.1 (1.7)
**Al^3+^**	pH 8	5.1 (0.1)	21.0 (0.7)	29.2 (0.8)	35.1 (1.4)	36.7 (1.4)	36.9 (1.4)	38.3 (1.7)	38.4 (1.8)
	pH 9	5.2 (0.3)	22.8 (0.0)	38.6 (1.3)	43.4 (1.2)	39.5 (1.6)	35.4 (1.6)	30.7 (1.4)	24.3 (0.9)
	pH 7	11.4 (0.9)	39.0 (1.4)	43.1 (1.9)	45.5 (1.1)	49.2 (1.2)	53.0 (1.8)	61.0 (1.5)	66.8 (2.2)
**Fe^3+^**	pH 8	5.0 (0.3)	32.2 (1.6)	41.7 (1.5)	43.6 (1.6)	44.4 (1.3)	45.5 (1.2)	49.4 (1.2)	52.3 (1.8)
	pH 9	1.1 (0.1)	3.1 (0.4)	5.3 (0.2)	10.7 (0.4)	26.4 (1.0)	35.2 (2.0)	41.3 (2.0)	42.7 (1.3)
				**Black Goji Extract**					
				[M^3+^]					
		2.5 μM	12.5 μM	25 μM	50 μM	125 μM	250 μM	750 μM	1500 μM
	pH 7	2.1 (0.2)	4.1 (0.7)	6.1 (0.3)	8.5 (0.4)	13.2 (1.0)	18.3 (1.3)	27.0 (2.2)	36.5 (1.8)
**Al^3+^**	pH 8	3.1 (0.1)	5.7 (0.5)	8.0 (0.6)	35.2 (1.7)	40.8 (2.2)	40.3 (1.9)	38.2 (1.9)	35.5 (2.5)
	pH 9	4.1 (0.7)	20.5 (0.9)	37.8 (1.8)	41.3 (1.5)	40.0 (1.7)	37.8 (2.2)	35.8 (1.3)	33.2 (1.5)
	pH 7	6.6 (0.8)	29.6 (1.2)	40.7 (1.8)	45.4 (1.8)	48.7 (1.9)	52.1 (2.2)	59.9 (2.9)	66.6 (3.1)
**Fe^3+^**	pH 8	0.5 (0.1)	3.7 (0.1)	19.6 (1.2)	32.6 (1.1)	35.3 (1.2)	35.9 (1.9)	40.1 (2.7)	45.1 (1.9)
	pH 9	1.1 (0.2)	2.6 (0.2)	5.6 (0.2)	10.1 (0.6)	25.2 (2.0)	30.9 (2.6)	43.9 (3.0)	48.0 (2.2)

## Supplementary Materials

The following are available online at https://www.mdpi.com/2304-8158/9/10/1426/s1, Table S1: Half-lives (hr) of purple potato petunidin derivatives chelated with Al^3+^ and Fe^3+^ ions at pH 7–9.
