# Supplementary material for: Metal Chelates of Petunidin Derivatives Exhibit Enhanced Color and Stability
Source: Foods. 2020 Oct 9;9(10):1426. doi: 10.3390/foods9101426 (PMC7599678; doi:10.3390/foods9101426)
Supplement: Supplementary file 1 [file foods-09-01426-s001.pdf]

**Supplementary Table 1:** Half-lives (hr) of purple potato petunidin derivatives chelated with $Al^{3+}$ and $Fe^{3+}$ ions at pH 7 - 9.

| | pH | $T_{1/2}$ (HR) [$M^{3+}$] 0 μM | 2.5 μM | 12.5 μM | 25 μM | 50 μM | 125 μM | 250 μM | 750 μM | 1500 μM |
|---|---|---|---|---|---|---|---|---|---|---|
| | 7 | 14.1(0.2) | 14.1(0.1) | 14.2(0.1) | 14.2(0.1) | 14.6(0.2) | 14.0(0.1) | 14.6(0.1) | 15.9(0.1) | 17.4(0.1) |
| **$Al^{3+}$** | 8 | 44.0(0.8) | 44.1(1.1) | 44.5(0.4) | 43.8(0.9) | 52.0(1.7) | 59.4(2.1) | 116.9(10.5) | 141.8(3.8) | 144.1(10.1) |
| | 9 | 12.9(0.1) | 13.6(0.4) | 15.4(0.2) | 19.5(0.2) | 19.1(0.1) | 19.7(1.8) | 18.9(0.9) | 19.5(0.1) | 21.7(2.1) |
| | 7 | 14.1(0.2) | 16.4(0.3) | 22.5(0.4) | 27.6(0.4) | 32.5(0.2) | 77.9(0.28) | 61.1(2.4) | 36.4(0.2) | 32.3(1.5) |
| **$Fe^{3+}$** | 8 | 44.0(0.8) | 43.9(0.3) | 43.5(1.2) | 41.5(1.5) | 98.5(7.2) | 43.1(2.8) | 41.9(3.0) | 36.6(5.3) | 35.1(5.6) |
| | 9 | 12.9(0.1) | 12.9(0.1) | 13.3(0.2) | 14.5(0.2) | 16.0(0.7) | 10.3(0.2) | 10.1(0.1) | 8.9(0.1) | 7.9(0.4) |
